# Distinct Intra‐Cohort Resource Utilization in Young‐of‐the‐Year Pikeperch ( *Sander lucioperca* ): Evidence From Diet and Isotopic Analysis

**DOI:** 10.1002/ece3.71973

**Published:** 2025-08-19

**Authors:** Million Tesfaye, Allan T. Souza, Marek Šmejkal, Tomáš Jůza, Zuzana Sajdlová, Vladislav Draštík, Luboš Kočvara, Radka Symonová, Travis B. Meador, Mojmír Vašek, Jan Kubečka

**Affiliations:** ^1^ Biology Centre of the Czech Academy of Sciences Institute of Hydrobiology České Budějovice Czechia; ^2^ Faculty of Fisheries and Protection of Waters, South Bohemian Research Centre for Aquaculture and Biodiversity of Hydrocenoses University of South Bohemia in České Budějovice Vodňany Czechia; ^3^ Faculty of Agriculture and Forestry Institute for Atmospheric and Earth System Research INAR, Forest Sciences, University of Helsinki Helsinki Finland; ^4^ Faculty of Science, University of South Bohemia in České Budějovice České Budějovice Czechia; ^5^ Institute of Soil Biology and Biogeochemistry Biology Centre of the Czech Academy of Sciences České Budějovice Czechia

**Keywords:** gut content analysis, intraspecific competition, ontogeny, piscivory, stable isotopes

## Abstract

Intraspecific competition is a fundamental selective force in animals, leading to various specializations that influence ecological interactions. Diet composition and trophic position at the early life stages substantially influence fish growth, survival, and recruitment success. Yet, most studies focus disproportionately on adult stages, leaving critical knowledge gaps in our understanding of early life history. To address this, we used young of the year (YOY) pikeperch ( *Sander lucioperca* ) as a model species and investigated the intraspecific interaction and degree of trophic partitioning between three intra‐annual cohorts (extremely small (ES), ordinary and piscivorous YOYs) using stable isotope (SI) and gut content analysis (GCA). Analysis of SI metrics unveiled that an ontogenetic diet shift was linked to increasing body size, leading to significant trophic niche variation among intra‐annual cohorts. The piscivorous cohort occupied the highest trophic position, followed by the ordinary and ES cohorts. There was no overlap in the isotopic niche between the intra‐annual cohorts, considering the 40% standard ellipse area. The GCA showed two distinct feeding patterns: the ES cohort exclusively consumed zooplankton, while the ordinary cohort had a more diverse diet, feeding on zooplankton and benthic macroinvertebrates. The piscivorous cohort (≥ 80 mm) predominantly fed on their conspecifics and YOY perch ( *Perca fluviatilis* ). Our study demonstrates that YOY pikeperch intra‐annual cohorts exhibit a broad size range and unique ontogenetic feeding patterns, with vital implications for population dynamics and ecological interactions. These differences are likely due to different hatching dates, environmental factors, and individual ability to become predatory. Furthermore, this work emphasizes the need for comparative studies to better understand trophic dynamics and uncover the ecological factors shaping the early life stages of fish.

## Graphical abstract

Young of the years intra‐annual cohorts showed a broad size range. Two distinct ontogenetic feeding patterns were observed. The isotopic niches of the three intra‐annual cohorts of pikeperch showed no overlap (considering 40% SEAc).

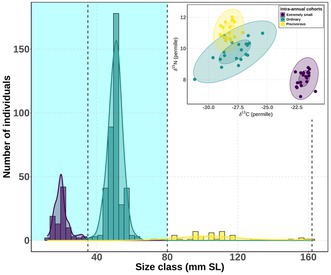

## 1 Introduction

Density‐dependent processes regulate individual fitness and population dynamics through intra‐ and interspecific competition and predation (Travis et al. 2023). These interactions often lead to changes in development, morphology, and habitat utilization and cause variations in trophic position within natural populations (Araújo et al. 2011; Bolnick et al. 2003). These variations may occur at any trophic level within a food web, eventually playing an important role in stabilizing the ecological dynamics (Guimerà et al. 2010). Competition for resources and predation can influence the degree of variation in the trophic niche position among individuals in a given population (Eklöv and Svanbäck 2006; Svanbäck et al. 2015; Svanbäck and Bolnick 2007). Particularly, intraspecific competition for food increases trophic niche variation among individuals by endorsing size‐based diet shifts, habitat differentiation, and dietary specialization (Jones and Post 2016). This may reduce dietary and habitat overlaps among conspecifics or sympatric species, potentially alleviating predation pressure (Schirmer et al. 2020; Schoener 1974).

The trophic niche positions vary largely between species and individuals of the same species at different life stages, and this has been especially studied in older age classes in fishes (Costa‐Pereira et al. 2018; Ru et al. 2022; Vejřík et al. 2023; Zhao et al. 2014). The extent and nature of these variations in the early life stages of a single species and how this relates to the trophic level of an individual in the food chain are far less well understood (Nunn et al. 2012; Sánchez‐Hernández et al. 2022; Westrelin et al. 2023).

As animals grow, body size varies, leading to changes in metabolic needs, resource utilization, and susceptibility to predation (Akin and Winemiller 2008; Bousseba et al. 2024; Guimerà et al. 2010; Uphoff et al. 2019). During the first year of life, most freshwater fish undergo an ontogenetic diet shift, from small‐sized zooplankton to other food sources such as plant material, macroinvertebrates, or fish (Huss et al. 2008; Qin et al. 2024; Sánchez‐Hernández 2019; Souza et al. 2020). For the latter group of fishes, the switch to piscivory is energetically beneficial for growth and development as it provides energy‐rich prey. Larger individuals in the group often make this dietary shift earlier than smaller individuals and achieve significantly faster growth, while smaller individuals that change their diet later may experience delayed growth (Frankiewicz et al. 1999; Persson and Brönmark 2008; van Densen et al. 1996). Small differences in the growth rate during the early phase of life can develop into large differences in the growth of individuals in later phases (Huss et al. 2008; Post 2003). Such differential growth has been observed in many piscivorous fish, including largemouth bass ( *Micropterus salmoides* ) (Zhao et al. 2014), walleye ( *Stizostedion vitreum* ) (Paradis et al. 2006; Uphoff et al. 2019), yellow perch ( *Perca flavescens* ) (Wu and Culver 1992), European perch (Heermann et al. 2007; Urbatzka et al. 2008), and pikeperch ( *Sander lucioperca* ) (Frankiewicz et al. 1999; Ginter et al. 2012a; Specziár 2005).

Pikeperch is a commercially valuable and ecologically important predatory fish in Eurasian water bodies; its role as a top predator is crucial for regulating prey populations and the structure and function of food webs (Jakubavičiūtė et al. 2024; Tesfaye et al. 2023). It is a piscivorous specialist that undergoes one or two distinct ontogenetic diet shifts during its early developmental stages, starting with zooplanktivory, switching to benthivory, and/or ending as piscivory (Specziár 2005; Vašek et al. 2018; Westrelin et al. 2023). This ontogenetic shift is highly dependent on the size of the young of the year (YOY), the availability of suitable prey, and environmental conditions, and could have either a positive or negative effect on the recruitment success of the populations (Galarowicz et al. 2006; Trochine et al. 2022). A better understanding of the processes behind ontogenetic dietary changes is important for identifying and predicting recruitment success. This has implications for overall population and ecological dynamics and demonstrates the importance of understanding the trophic ecology of pikeperch in the early life stages for effective fisheries management and conservation of populations in an ever‐changing environment.

Gut content analysis (GCA) and stable isotope analysis (SIA) are the two common methods for determining the structure of the food web, trophic interactions, and essential energy sources of consumers (Davis et al. 2012; Vašek et al. 2018). Gut content analysis (GCA) can provide comprehensive taxonomic information on recently ingested prey with its abundance and diversity (Akin and Winemiller 2008; Cobain et al. 2019; Gutmann Roberts and Britton 2018). At the same time, SIA captures resource utilization and trophic interactions and ultimately reflects feeding behavior and assimilated diet over time (Jackson et al. 2011; McCue et al. 2020; Šmejkal et al. 2024). Integrating these two complementary approaches improves our understanding and provides clearer information about dietary shifts, trophic ecology, and the co‐existence of multiple size groups during the early life stages of fish.

The feeding ecology and ontogenetic diet shifts of YOY pikeperch have indeed been extensively studied (Frankiewicz et al. 1999; Ginter et al. 2012a, 2011; Persson and Brönmark 2008). A number of studies also investigated mechanisms beyond the ontogenetic diet shift, resulting in individual size variation in related fish species, such as perch (Heermann et al. 2007; Huss et al. 2008; Urbatzka et al. 2008) and walleye (Galarowicz et al. 2006; Schoenebeck et al. 2024; Uphoff et al. 2019). However, many of these studies lack empirical evidence and are primarily experimental. Furthermore, in addition to the distinct intra‐annual cohorts of YOY pikeperch previously identified (ordinary pelagic and littoral piscivorous) (Frankiewicz et al. 1999, 1996; van Densen 1985; van Densen et al. 1996), a newly described extremely small YOY appears to be abundant in freshwater systems (Jůza et al. 2013; Tesfaye et al. 2024). This suggests the presence of multiple intra‐annual cohorts in some years, highlighting the need for further investigation into their dietary adaptations, ontogenetic diet shifts, and intra‐specific interactions among YOY individuals.

Our study aims to explore intraspecific interactions and the degree of trophic partitioning among conspecifics of YOY pikeperch using GCA and SIA. Specifically, we hypothesized that (i) the diet composition of YOY pikeperch differs among intra‐annual cohorts, with larger individuals exhibiting a broader dietary niche and higher trophic positions due to their increased size and access to a broader range of prey. In comparison, smaller individuals are expected to show a narrower dietary niche, reflecting size‐related constraints on prey selection, and we expect a certain degree of overlap in the isotopic position. Further, we hypothesized that (ii) intraspecific competition among the YOY individuals drives trophic niche partitioning, facilitating coexistence and promoting individual specialization.

## 2 Materials and Methods

### 2.1 Study Site

**FIGURE 1:**
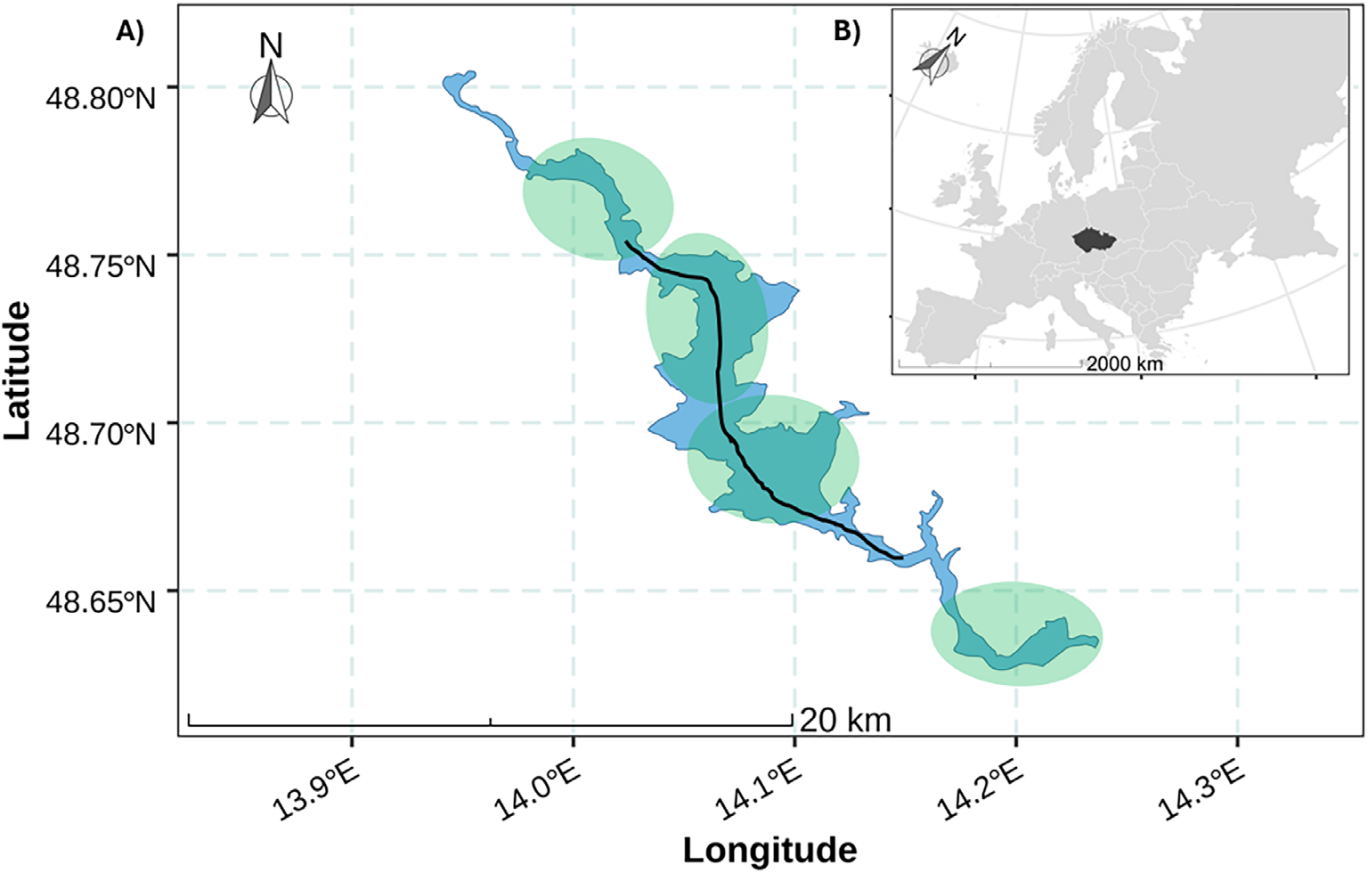
Map of the study area in the Lipno Reservoir, Czechia. (A) Sampling locations within the reservoir. Light green shaded circles indicate the sites for benthic gillnet sampling, while the black line represents the longitudinal transect for pelagic trawling. (B) Map showing the location of Czechia (highlighted in black) within Europe.

Lipno Reservoir is a shallow, eutrophic, and large water body in the South Bohemian region of Czechia (Figure 1). It has a water volume of 306 million m^3^, a surface area of 46.5 km^2^, a maximum depth of 22 m, a mean depth of 6.6 m, a shoreline length of 115 km, and a mean water residence time of 0.6 years (Krolová et al. 2013, 2012). The reservoir was constructed between 1952 and 1959 by damming the Vltava River with the aim of providing multifunctional purposes: hydropower generation, flood protection, flow augmentation, and recreational activities (Krolová et al. 2012). Despite its eutrophic classification, mean water transparency, as measured by Secchi depth, is around 2 m, and mean chlorophyll‐a concentration is 18 μg L^−1^(Tesfaye et al. 2023). The system is also subject to significant water level fluctuations driven by its management for hydropower and flood control (Krolová et al. 2013). Previous studies have shown that the fish community in the reservoir consists mainly of cyprinids (white bream *Blicca bjoerkna* , bleak *Alburnus alburnus* , roach *Rutilus rutilus* and freshwater bream *Abramis brama* ). The most common predatory species include the adult European perch *Perca fluviatilis* , pikeperch, Northern pike *Esox lucius* , and the European catfish *Silurus glanis* (De Moraes et al. 2023).

### 2.2 Field Sampling and Sample Processing

Fish were sampled at the end of August 2023 using gillnets and trawling. These two methods were opted to account for the two main habitats of the reservoir, benthic and pelagic. Piscivorous YOY inhabit the benthic part of the reservoir and cannot be effectively sampled using pelagic trawling (Symonová et al. 2024). To sample this intra‐annual cohort, we employed multimesh gillnets with 12 mesh sizes, following the European standard EN 14757, supplemented by four additional larger mesh sizes (70, 90, 110, and 135 mm) (for details of the gillnet sampling, see Šmejkal et al. 2015). Gillnets were set before sunset and retrieved after sunrise to capture the peak periods of fish activity (Prchalová et al. 2010). All captured fish were identified to the species level. Their standard length (SL) was measured to the nearest millimeter. Following Symonová et al. (2024), we defined piscivorous YOY as individuals with a standard length (SL) of ≥ 80 mm; the age of these fish (0+) was then further confirmed through otolith readings. These individuals were then selected for further analysis. In addition, their abundance was expressed as catch per unit of effort (CPUE), that is, individuals caught per 1000 m^2^ of multimesh gillnets per night. Pelagic trawling with a fixed frame of a 3 × 3 = 9 m^2^ mouth opening (as described in Jůza and Kubečka 2007) was employed to sample pelagic YOY pikeperch. The mesh size of the trawl was 6.5 mm knot to knot in the belly and 4 mm in the cod end. The lower part of the frame was equipped with two 15 kg iron slides to keep the trawl weighted down and protect it from potential contact with the bottom. An accessory boat was attached to the upper rim to ensure the trawl remained at the desired depth (Jůza et al. 2018). A rope was attached between the boat and the frame for deeper tows, so we sampled the layers of 0–3, 3–6, 6–9, and 9–12 m. The trawl was towed at night 100 m behind the boat at approximately 1 m/s^−1^. Each haul lasted 10 min. Twenty‐eight hauls were conducted, covering all available depth strata at a given location. The trawl was kept on a slightly curved path during all hauls to avoid disturbing the sampling areas by the towing vessel. The SL of each fish was measured to the nearest millimeter. A sample of dorsal muscle was taken for fish with an SL above 40 mm. For fish with an SL below 40 mm, the entire gutted body, excluding the head (due to the small size of the sampled individuals), was stored at −20°C until further processing for stable isotope analysis. The digestive tracts of all pikeperch captured were dissected and preserved in a 10% formaldehyde solution for later GCA.

We explored the structure of size distribution of YOY pikeperch sampled using trawling and benthic gillnets and performed a multimodality test following the approach described in Tesfaye et al. (unpublished manuscript). As outlined in Tesfaye et al. (unpublished manuscript), the size distribution of YOY pikeperch can exhibit unimodal, bimodal, or trimodal patterns, with trimodality occurring less frequently. Therefore, a multimodality test was necessary. Initial detection of multimodal size distributions was conducted using the dip.test function from the diptest R package (Maechler 2024). Additionally, trimodality tests were performed using the modetest function from the multimode R package (Ameijeiras‐Alonso et al. 2021).

### 2.3 Gut Content Analysis

In the laboratory, the digestive tracts of 60 individuals were dissected and examined under a stereomicroscope. Prey items were identified to the lowest practical taxonomic level and counted, and their volumetric contribution to the total stomach content was visually estimated (Hyslop 1980). When possible, characteristic remains (e.g., fish bones and invertebrate exoskeleton fragments) were used to identify partially digested prey items.

### 2.4 Stable Isotope Analysis

Sixty YOY pikeperch samples, the same individuals analyzed for gut content analysis (GCA), were also subjected to stable isotope analysis (SIA) (Table 1). The muscle samples or eviscerated bodies without heads in the case of the extremely small intra‐annual cohort were dried at 60°C for 48 h, then pulverized into a fine powder, homogenized with a mixer mill MM 200 (Retsch GmbH, Haan, Germany), and filled into tin capsules for weighing, which were then packed for isotope analyses. The stable isotopic ratios of carbon and nitrogen in the pikeperch samples were determined at the Soil and Water Research Infrastructure, Biology Centre of the Czech Academy of Sciences (České Budějovice, Czechia) using a MAT253 Plus isotope ratio mass spectrometer coupled to an elemental analyzer (EA Isolink CNSOH) via a Conflo IV interface (all equipment from Thermo Scientific, Bremen, Germany). Samples were analyzed together with the international reference material IAEA‐600 and an in‐house fish muscle working standard. Results were reported in conventional delta notation, normalized to the international reference scale (δ^13^C‐VPDB and δ^15^N‐air ‰). Repeated measurements of the reference standards indicated that the precision was < 0.2‰ and < 0.3‰ for δ^13^C and δ^15^N, respectively.

**TABLE 1:** Summary from randomly selected samples of stable isotope and gut content analysis from each of the three intra‐annual cohorts of pikeperch ( *Sander lucioperca* ) from Lipno Reservoir, Czechia, including sample size (*n*) and standard length (SL) statistics (mm).

Intra‐annual cohorts	No. of individuals (*n*)	Mean SL (mm)	SD (mm)	Size range SL (mm)
Extremely small	20	19.60	2.11	13.50–24.00
Ordinary	20	51.90	6.66	38.00–72.00
Piscivorous	20	99.00	11.33	81.00–115.00

### 2.5 Statistical Analyses

To examine differences in the volumetric proportions of prey categories in the digestive tracts of individuals between intra‐annual cohorts, we conducted a Permutational Multivariate Analysis of Variance (PERMANOVA) using the adonis2 function from the vegan R package (Oksanen et al. 2007). This was followed by post hoc pairwise PERMANOVA comparisons using the pairwise.adonis function from the pairwiseAdonis R package (Martinez Arbizu 2020) to identify specific differences between the intra‐annual cohorts.

For stable isotope analysis, PERMANOVA was also performed to examine whether the occupied isotopic niches differed significantly between the intra‐annual cohorts. After confirming significance, post hoc pairwise PERMANOVA comparisons were performed to determine whether the observed differences were due to the δ^13^C or δ^15^N values (Balzani et al. 2024).

The isotopic niche size of each intra‐annual cohort of YOY pikeperch was estimated in δ^13^C–δ^15^N space using standard ellipse areas (40% EA). This metric provides a bivariate measure of the distribution of individuals in isotopic space, with the ellipse including the mean 40% of the data, representing the typical resource utilization of the intra‐annual cohort of YOY (Jackson et al. 2011). Additionally, the 95% ellipse area (95% EA) was plotted, serving as a bivariate equivalent of the 95% confidence interval encompassing 95% of the sample (Layman and Allgeier 2012).

The percentage of overlap between intra‐annual cohorts was calculated as two overlapping ellipse areas (Bayesian 40% or 95% EA) divided by the sum of the ellipse areas of the two annual cohorts intra‐annual cohorts multiplied by 100, based on the equation of Stasko et al. (2015). This percentage was computed for each posterior draw of the Bayesian estimates of overlap and ellipse areas, producing a distribution with 1000 iterative draws using the SIBER package (Jackson et al. 2011). Furthermore, the directional pairwise probability of an individual from one intra‐annual cohort falling into the niche of another one was estimated at the 95% Bayesian ellipse area. This estimation was performed using the nicheROVER package (Lysy et al. 2023) with a Monte Carlo simulation of 10,000 iterations.

Based on Layman et al. (2007), six isotopic niche metrics were calculated to assess the potential difference between intra‐annual cohorts: the δ^15^N range (NR) and the δ^13^C range (CR) as differences between the most enriched and the most depleted individual, the mean Euclidean distance of each individual from the centroid of the δ^13^C–δ^15^N values (CD), the mean nearest neighbor distance in δ^13^C–δ^15^N space (NND) and its standard deviation (SDNND). Additional isotopic niche metrics were computed to quantify and compare trophic niche dimensions among the three intra‐annual cohorts: Total Area (TA), which represents the total area of the convex hull encompassing all data points in δ^13^C–δ^15^N isotopic space and provides a measure of the overall niche width; Standard Ellipse Area (SEA), which estimates the core isotopic niche area by excluding outliers and is less sensitive to sample size compared to TA; and Corrected Standard Ellipse Area (SEAc), a bias‐corrected version of SEA adjusted for small sample sizes to ensure robust comparisons across groups (Jackson et al. 2011).

All statistical analyses and figures were generated using R version 4.4.2 (R Core Team 2023) with the R packages tidyverse (Wickham et al. 2019), SIBER (Jackson et al. 2011) and nicheROVER (Lysy et al. 2023).

## 3 Results

### 3.1 Size Distribution of Young of the Year Pikeperch

**FIGURE 2:**
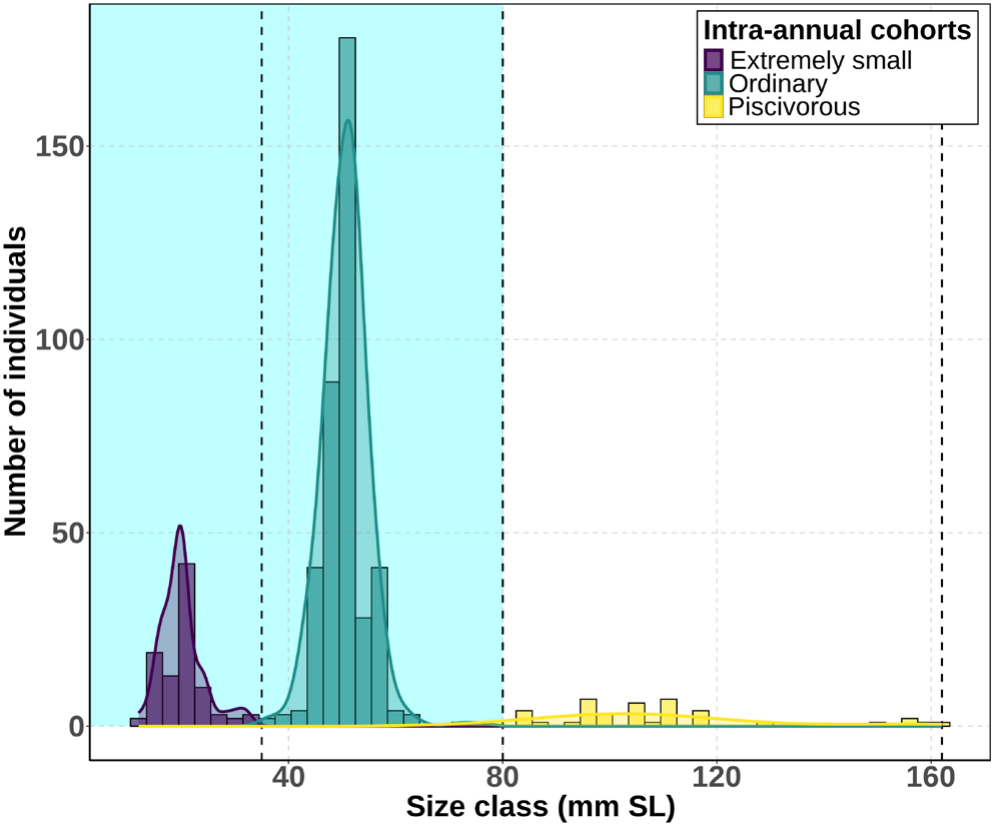
Length frequency of all young‐of‐the‐year (YOY) pikeperch ( *Sander lucioperca* ) captured by trawl (shaded by light blue) and benthic gillnets (shaded by white) in LipnoReservoir, Czechia. The dotted line indicates the cutoff length among the intra‐annual cohorts.

The size distribution analysis of YOY pikeperch, sampled by trawling and benthic gillnets, revealed a clear trimodal size structure (*D* = 0.055, *p* < 0.001; Figure 2), providing strong evidence for the presence of three distinct intra‐annual cohorts in the size distribution. Pelagic intra‐annual cohorts, that is, the ES and ordinary fingerlings, were generally more abundant than littoral piscivores in August of their first year of life (Figure 2).

### 3.2 Gut Content Analyses

**FIGURE 3:**
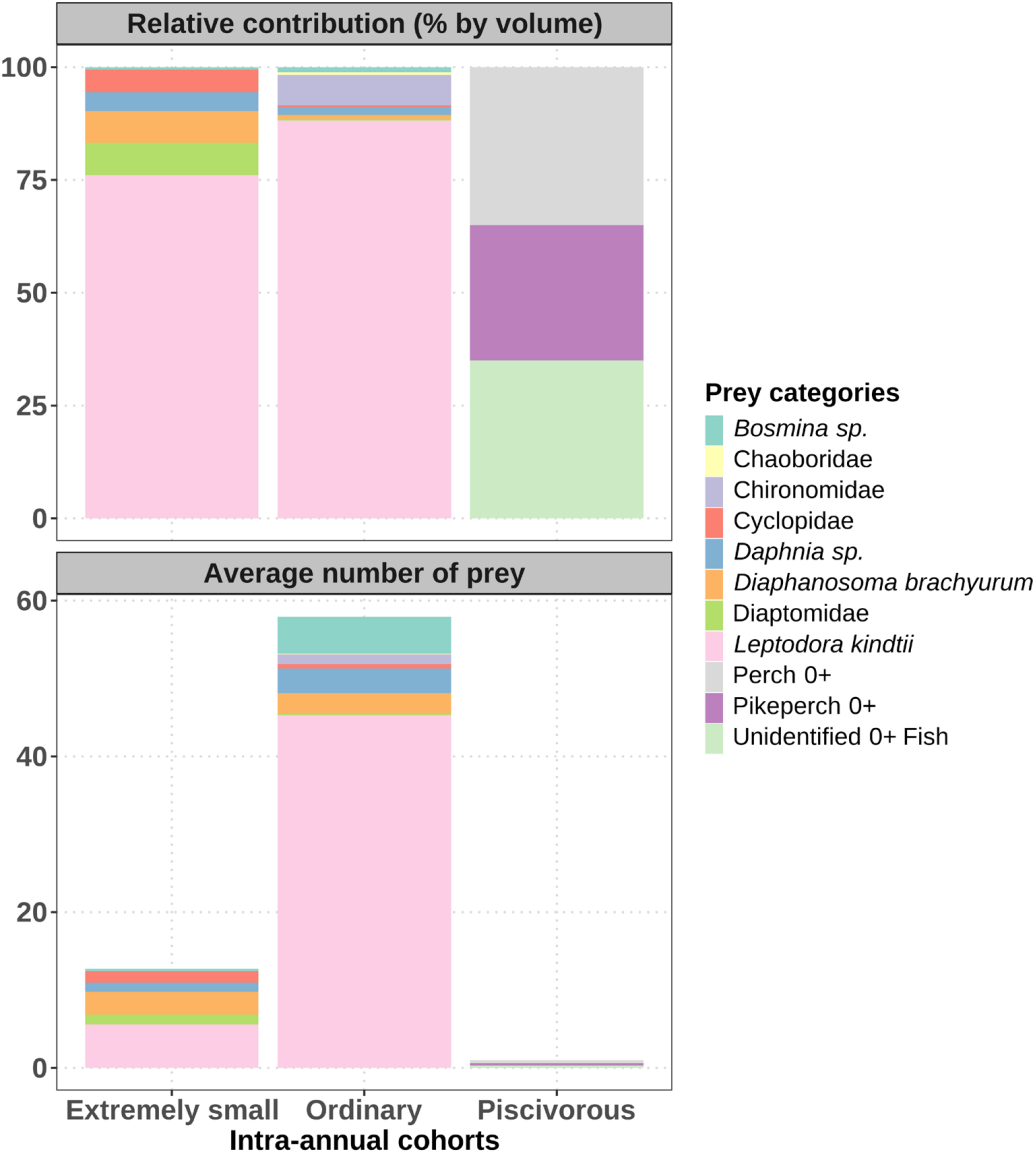
The average number of prey and relative volumetric proportions (%) of major prey items identified in the stomachs of the three intra‐annual cohorts of young‐of‐the‐year (YOY) pikeperch ( *Sander lucioperca* ) during the study period in Lipno Reservoir, Czechia.

A total of 60 non‐empty stomachs were analyzed, and the data showed distinct ontogenetic dietary shifts and differential use of prey resources by YOY pikeperch conspecifics. The statistical results showed a strong significant difference in the prey composition among the three intra‐annual cohorts (PERMANOVA: *R* ^2^ = 0.565, *F*_3,35_ = 15.165, *p* < 0.001). Specifically, the extremely small intra‐annual cohort differed significantly from the ordinary (post hoc pairwise PERMANOVA: *F* = 267.57, *p* < 0.001, *R* ^2^ = 0.87) and piscivorous intra‐annual cohorts (post hoc pairwise PERMANOVA: *F* = 1033.89, *p* < 0.001, *R* ^2^ = 0.96). The ordinary intra‐annual cohort differed significantly from the piscivorous intra‐annual cohort (F‐mod = 15.39, *p* < 0.001, *R* ^2^ = 0.30). Extremely small YOY pikeperch (ES, < 37.5 mm) fed exclusively on zooplankton such as *Leptodora kindtii* , *Diaphanosoma brachyurum* , *Daphnia* sp., Diaptomidae, and Cyclopidae, whereas the ordinary YOY pikeperch consumed both zooplankton, mainly *L. kindtii* , and benthic macroinvertebrates, such as chironomid larvae. On the other hand, piscivorous YOY pikeperch preyed nearly exclusively on YOY fish, including pikeperch, perch, and other species (Figure 3). The large cladoceran *L. kindtii* was the most important food for ES and ordinary intra‐annual cohorts, contributing more than 75% of the diet volume.

### 3.3 Stable Isotope Analyses

#### 3.3.1 Differences in Isotopic Niches Between YOY Intra‐Annual Cohorts

**FIGURE 4:**
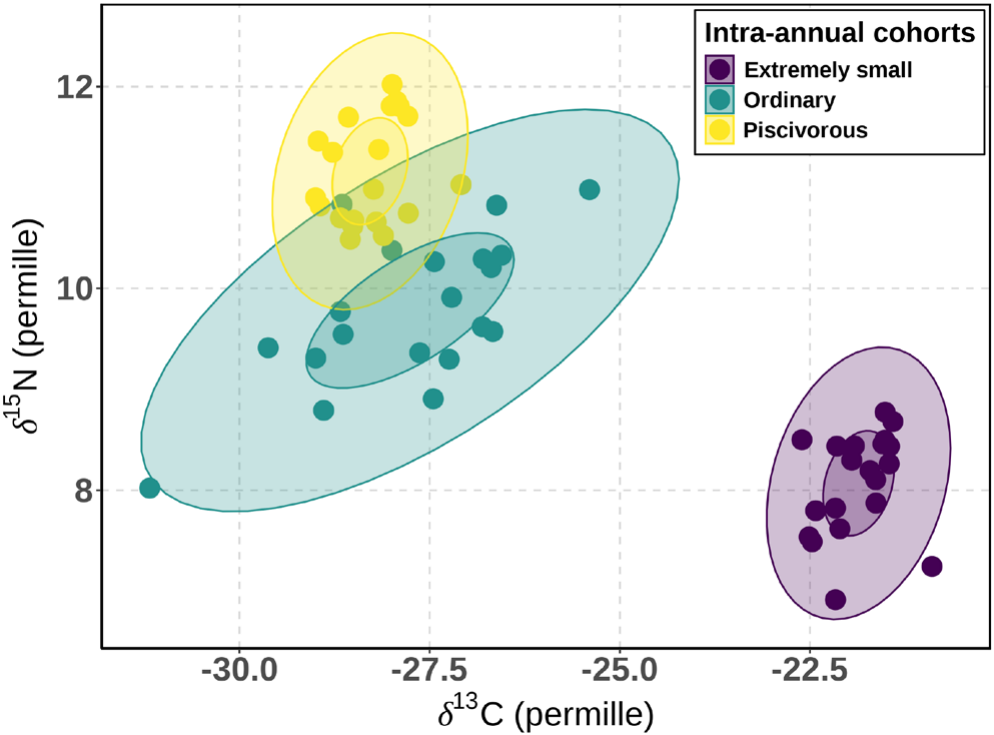
Isotopic niches of the three intra‐annual cohorts of pikeperch ( *Sander lucioperca* ) in Lipno Reservoir, Czechia, 40% standard ellipse area for three intra‐annual cohorts (darker shading) and 95% ellipse area (lighter shading). Solid lines = ellipse areas.

The isotopic niches of individuals belonging to the three intra‐annual cohorts showed significant differences in their δ^15^N (PERMANOVA: *F*_2,57_ = 130.77, *R* ^2^ = 0.82, *p* < 0.001) and δ^13^C (PERMANOVA: *F*_2,57_ = 343.65, *R* ^2^ = 0.92, *p* < 0.001) values. Individuals from the piscivorous intra‐annual cohort had the highest δ^15^N values, followed by the ordinary and extremely small cohorts, and the three intra‐annual cohorts differed significantly in their mean δ^15^N values (Table 2, Figure 4). In terms of δ^13^C, extremely small YOY pikeperch had the highest values and differed significantly from individuals belonging to the ordinary (post hoc pairwise PERMANOVA: *F* = 350.34, *R* ^2^ = 0.90, *p* < 0.001) and piscivorous (post hoc pairwise PERMANOVA: *F* = 1860.03, *R* ^2^ = 0.97, *p* < 0.001) intra‐annual cohorts, while there was no significant difference observed between piscivorous and ordinary intra‐annual cohorts (post hoc pairwise PERMANOVA: *F* = 2.74, *R* ^2^ = 0.067, *p* > 0.05; Table 2, Figure 4).

**TABLE 2:** Results of post hoc pairwise PERMANOVA analysis.

Comparison between intra‐annual cohorts	Isotopic element	F‐model	*R* ^2^	*p*
Extremely small—Ordinary	δ^15^N	70.48	0.65	< 0.001***
	δ^13^C	350.34	0.90	< 0.001***
Extremely small—Piscivorous	δ^15^N	355.54	0.90	< 0.001***
	δ^13^C	1860.0	0.98	< 0.001***
Ordinary—Piscivorous	δ^15^N	45.80	0.54	< 0.001***
	δ^13^C	2.73	0.067	0.108

*Note:* Significant differences were detected in both δ^15^N and δ^13^C values among the intra‐annual cohorts (extremely small, ordinary, and piscivorous YOYs). Pairwise comparisons were conducted to evaluate the differences between the three groups. Significance: ***p < 0.001, **p < 0.01, *p < 0.05, = not significant.

#### 3.3.2 Niche Sizes and Overlaps

**FIGURE 5:**
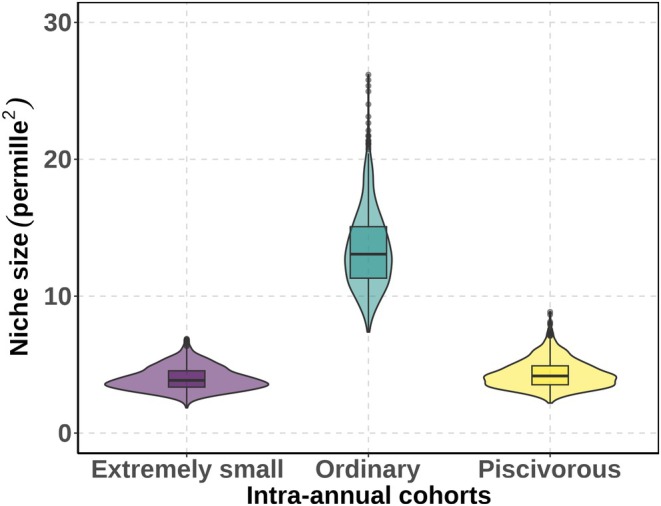
Posterior distribution of niche size estimates for the three young‐of‐the‐year (YOY) pikeperch ( *Sander lucioperca* ) intra‐annual cohorts in Lipno Reservoir, Czechia. Niche size is calculated from Bayesian posterior samples of 𝜇 (mean) and Σ (covariance matrix) using a 95% credible interval, modeled with nicheROVER package (Lysy et al. 2023).

The niche metrics varied between the three intra‐annual cohorts. The isotopic niche size of the extremely small and piscivorous intra‐annual cohorts was relatively narrow. In contrast, the ordinary intra‐annual cohort exhibited a larger isotopic niche (Figure 5). The niche width (proxied by TA, SEA and SEAc) of the ordinary intra‐annual cohort is more than three times higher than that of the piscivorous and extremely small intra‐annual cohorts (Table 3). The metrics for niche dispersion (i.e., CD, NND and SDNND) also showed similar patterns and were all higher in the ordinary intra‐annual cohort (Table 4).

**TABLE 3:** Summary of isotopic niche metrics for the three intra‐annual cohorts of young‐of‐the‐year pikeperch ( *Sander lucioperca* ) from Lipno Reservoir, Czechia.

Isotopic niche metrics	Extremely small	Ordinary	Piscivorous
TA	2.19	7.47	1.79
SEA	0.70	2.38	0.76
SEAc	0.74	2.51	0.80

*Note:* Metrics include TA (Total Area), SEA (Standard Ellipse Area), and SEAc (Corrected Standard Ellipse Area), which quantify and compare trophic niche dimensions in δ^13^C–δ^15^N isotopic space.

**TABLE 4:** Layman's metrics for isotopic niches of extremely small, ordinary, and piscivorous young of the year pikeperch ( *Sander lucioperca* ) intra‐annual cohorts from Lipno Reservoir, Czechia.

Intra‐annual cohorts	CR	NR	CD	NND	SDNND
Extremely small	1.70	1.86	0.60	0.22	0.23
Ordinary	5.78	2.96	1.27	0.50	0.46
Piscivorous	1.91	1.53	0.66	0.21	0.17

Abbreviations: CD, mean distance to the centroid; CR, δ^13^C range; NND, mean nearest neighbor distance; NR, δ^15^N range; SDNND, standard deviation of nearest neighbor distance.

Bayesian overlap analysis (40% of ellipse area) showed no overlap between isotopic niches of intra‐annual cohorts. However, a minor overlap in the isotopic niche between the ordinary and piscivorous intra‐annual cohorts was observed (1.9% and 0% at 95% and 40% ellipse area, respectively). The Bayesian directional niche overlap analysis results suggested that the probability of individuals (*α* = 95%) of the ordinary intra‐annual cohort entering the piscivorous intra‐annual cohort niche was 10.3%; vice versa, it was 31.0% (Table 5).

**TABLE 5:** Ellipse areas (EA) and isotopic niche overlaps for 40% and 95% of niche regions.

Intra‐annual cohorts	40% EA	40% EA overlap			95% EA	95% EA overlap			P of ind. overlap at 95%		
		Extremely small	Ordinary	Piscivorous		Extremely small	Ordinary	Piscivorous	Extremely small	Ordinary	Piscivorous
Extremely small	0.76	—	0.00	0.00	4.44	—	0.00	0.00	NA	0.00	0.00
Ordinary	0.26	0.00	—	0.00	15.01	0.00	—	1.93	0	NA	10.28
Piscivorous	0.82	0.00	0.00	—	4.80	0.00	1.93	—	0	31.22	NA

*Note:* The EA and overlaps were calculated using Bayesian posterior probability distributions with the R package SIBER (Jackson et al. 2011). The directional probability of individual overlap at 95% confidence (P of ind. overlap at 95%) into the niche of other intra‐annual cohorts was calculated using the R package nicheROVER (Lysy et al. 2023).

## 4 Discussion

Both GCA and SIA suggested a clear ontogenetic dietary shift and isotopic niche segregation between YOY pikeperch in the studied reservoir. So far, limited information on resource use and partitioning among YOY pikeperch individuals has been available (Persson and Brönmark 2008; Specziár 2005). The current study is also the first to apply the SIA approach and the conventional GCA method to characterize the dietary niches of the co‐existing pikeperch intra‐annual cohorts. Thus, the findings of this study provide important insights into the trophic ecology of predatory fish populations and their roles in freshwater food webs.

### 4.1 Size Distribution and Ontogenetic Dietary Shift

The distribution of the size of YOY pikeperch showed a clear trimodality. Indeed, previous studies in the same ecosystem found the occurrence of trimodality in the YOY pikeperch in some of the years (Tesfaye et al. unpublished manuscript). Several factors have been suggested by earlier studies that could lead to the formation of a broad size distribution, eventually leading to a multi‐modal size distribution: (i) asynchronous hatching or spawning period (Huss et al. 2007; Post 2003; Jůza et al. 2013), (ii) prey availability and suitability (Tesfaye et al. 2024; Zorn et al. 2020), and (iii) environmental variability (climatic conditions during the first growing season) (Deboer et al. 2013; Zhang et al. 2017). All these factors together may lead to size‐dependent foraging and competition, as well as cannibalism/piscivory (Frankiewicz et al. 1999; Huss et al. 2008).

Our study confirmed the hypothesis (H1) that the development of a broad size distribution was coupled with partitioning in the diet between YOY pikeperch intra‐annual cohort and differences in hatching time. Indeed, it is well documented that pikeperch spawns in spring, typically starting in February and ending in June near its southern and northern distribution limits, respectively (Lappalainen et al. 2003). However, our recent investigation of ES fingerlings, using daily otolith ring analysis, revealed that summer spawning also occurs (unpublished data), which could be an additional factor that contributed to the observed broad range of size distribution. We observed two distinct ontogenetic diet shifts in the YOY pikeperch. In the first stage, ES intra‐annual cohort (10–34 mm) fed mainly on larger crustaceans such as *L. kindtii* , *D. brachyurum* , *Daphnia* sp., Diaptomidae, and Cyclopidae. Previous studies have made similar observations in which similarly sized YOY pikeperch fed exclusively on larger zooplankton (Specziár 2005).

In the second stage, ordinary YOY, with an intermediate size of 37–80 mm, continued feeding on planktonic crustaceans and consumed benthic macroinvertebrates such as Chironomidae. Previous studies have similarly reported relatively high consumption of chironomids by YOY pikeperch of this size class in various water bodies (Araújo et al. 2011; Kakareko 2002; Peterka et al. 2003; Specziár 2011; Verreth and Kleyn 1987). Studies on related species (i.e., walleye) demonstrated that intermediate‐size YOY fed on large calanoids and gained an energetic advantage, promoting improved growth and development of sufficient gape size to transition to piscivory (Uphoff et al. 2019). It is important to note that *L. kindtii* accounts for more than 75% of the diet of both pelagic groups (ES and ordinary intra‐annual cohorts), which is consistent with earlier reports of pelagic YOY pikeperch feeding predominantly on *L. kindtii* (Ginter et al. 2012b; Peterka et al. 2003; Specziár 2005).

The subsequent ontogenetic diet shift to piscivory was linked with the intra‐annual cohort size class ranging from 80 to 160 mm, and GCA revealed that they primarily consumed their conspecifics and YOY perch. Previous studies have also shown that larger YOY pikeperch individuals inhabiting littoral habitats fed mainly on fish prey (Frankiewicz et al. 1999; Ginter et al. 2012a). Prey fish served as the main food resource for this intra‐annual cohort and may have contributed to fast growth; this phenomenon was also described for other percid species, such as YOY walleye and perch (Huss et al. 2008; Uphoff et al. 2019). Over one‐third of the prey fish ingested by piscivorous YOY pikeperch were unidentifiable at the species level, despite pikeperch swallowing their prey without chewing them. Therefore, it is possible that the contribution of percid fish to the diet of the piscivorous YOY pikeperch intra‐annual cohort may be even higher.

The shortage of potential prey fish can delay the dietary shift to piscivory. This ultimately leads to a reduction in the abundance of piscivorous individuals and, in turn, affects the population size of the adult pikeperch because the dietary shift plays a key role in accelerating growth and reducing winter mortality due to starvation and size‐related predation (Huss et al. 2008). Based on our GCA result and previous studies, the co‐occurrence of the three intra‐annual cohorts in the same year was mainly determined by the characteristics and availability of a prey supply of suitable size and optimal environmental conditions (Frankiewicz et al. 1996; van Densen et al. 1996). The size spectra of a potential prey must overlap with the prey window defined by the predator's morphological, physiological, and behavioral capabilities (Galarowicz et al. 2006; Graeb et al. 2005; Persson and Brönmark 2002). If there is a mismatch between the size spectra of the predator and its prey, relatively fewer YOY pikeperch individuals switch to piscivory, and a majority stay either as ES or ordinary YOY (Frankiewicz et al. 1996; Sutela and Hyva 2002; van Densen 1985). Alternatively, there is a high probability that three intra‐annual cohorts co‐exist if water temperatures in spring and summer are optimal and there is a match in size spectra between predators and their prey (Bopp et al. 2023; Brandt et al. 2022; Sánchez‐Hernández 2024).

### 4.2 Trophic Interactions Between ES, Ordinary, and Piscivorous Intra‐Annual Cohorts

The ES intra‐annual cohort had higher δ^13^C values than ordinary and piscivorous intra‐annual cohorts. This pronounced difference in δ^13^C values between ES and ordinary fingerlings is due to the shorter lifetime and feeding period in ES fingerlings. This intra‐annual cohort likely originated from later spawning events, and because small organisms have a higher metabolic rate and, thereby, faster isotopic turnover in their tissues, their isotopic composition integrates dietary intake over a relatively shorter time frame compared to larger individuals. Zooplankton δ^13^C values typically increase seasonally due to enhanced primary production, which reduces the discrimination against the heavier carbon isotope by algae (Woodland et al. 2012; Yoshioka et al. 1994). The higher δ^13^C values observed in ES fingerlings in the current study likely reflect their reliance on zooplankton during late summer, when the base of the pelagic food chain had very probably elevated δ^13^C values. In contrast, the lower δ13C values of the earlier plankton bloom are integrated into the diet of the other two intra‐annual cohorts (Hobson et al. 2012; Wada 2009).

Relative δ^15^N values are an indicator of the trophic position of the species (Post 2002; Westrelin et al. 2023), and these differed significantly between the three intra‐annual cohorts. The piscivorous intra‐annual cohort had higher δ^15^N values, indicating that this group occupied a higher trophic position, followed by the ordinary and ES intra‐annual cohorts, which occupied the intermediate and lower trophic positions, respectively. The observed increase in trophic position with size is consistent with previous studies (Linzmaier et al. 2018; Vašek et al. 2018; Westrelin et al. 2023), and corresponds to the dietary shift from zooplanktivores to benthivores and finally to piscivores, which can reduce intraspecific competition between intra‐annual cohorts and increase the magnitude of niche partitioning between intra‐annual and intra‐annual cohorts (Ginter et al. 2011; Svanbäck and Bolnick 2007; Vašek et al. 2018; Westrelin et al. 2023). Similar observations have been reported in YOY northern pike, where the trophic position increased due to a shift towards greater importance of littoral prey, and individuals could differ by up to two trophic levels, reducing intraspecific competition (Beaudoin et al. 1999).

### 4.3 Intraspecific Niche Partitioning Between ES, Ordinary, and Piscivorous Intra‐Annual Cohorts

Based on our results, the ordinary intra‐annual cohort showed a wider isotopic niche area (SEAc 40% and 95%) and increased trophic diversity among individuals (CD) than the ES and piscivorous intra‐annual cohorts, which could be related to their high abundance among intra‐annual cohorts. As reflected in our GCA result, another possible explanation could be that the ordinary intra‐annual cohorts had a more diverse diet, being in a transitional phase from zooplanktivores to piscivores (Symonová et al. 2024), which could have increased the size of the isotopic niche. Similar to our study, a previous study has reported an increase in isotopic niche divergence due to higher densities in YOY pikeperch, indicating a possible intraspecific competition in which the pikeperch adjusts its trophic niche according to the density of its conspecifics (Westrelin et al. 2023).

The isotopic niches of the three intra‐annual cohorts of pikeperch showed no overlap (considering 40% SEAc) or only a moderate degree of overlap, that is, 0%–31% (considering 95% SEAc). The ordinary intra‐annual cohort did not overlap with the ES intra‐annual cohort but showed moderate overlap with the piscivorous sub‐cohort. These results support the hypothesis that intraspecific competition among the three YOY intra‐annual cohorts drives trophic niche partitioning, facilitating coexistence and promoting individual specialization.

The foregoing theory suggests that individuals living together might consume different food resources, leading to individual diet variation for several reasons (Araújo et al. 2011; Stephens and Krebs 1986). Our study has shown that ontogenetic diet shifts and body size variation cause the marked differences in diet and isotopic niche between intra‐annual cohorts of YOY pikeperch. The ontogenetic dietary shift of YOY pikeperch from zooplankton to macroinvertebrates and then to fish reflects size‐dependent changes in prey preferences. Studies have shown that an increase in size due to better growth rates can be caused by a switch to more profitable food resources (Sánchez‐Hernández 2019). As demonstrated in our study, individuals that become piscivores are almost three times larger than those of their ES conspecifics and twice as large as those of the ordinary sub‐cohort, and individuals of the ordinary intra‐annual cohort are also twice as large as those of their ES conspecifics that may not switch to piscivory (Tesfaye et al. unpublished manuscript). Furthermore, differences in habitat utilization and resource competition exacerbate these size differences, with pelagic and littoral groups exploiting distinct prey types (Franz et al. 2002; Trochine et al. 2022). These factors collectively stress the dynamic foraging ecology of YOY pikeperch in their first year of life and have huge implications for population and community ecological dynamics (Nunn et al. 2012).

### 4.4 Implications for Recruitment Success, Population and Ecological Dynamics

The recruitment success of many fish species is positively related to their size (growth) and successful dietary shift at an early stage of their life (Fuiman and Higgs 1997; Nunn et al. 2010), which may ultimately influence the lifetime fitness of individual fish, population dynamics, and other size‐dependent processes (Huss et al. 2013; Post 2003). Persson and Brönmark (2002) pointed out that the recruitment success of pikeperch depends mainly on the timing of the ontogenetic switch to the piscivorous intra‐annual cohort in the first year. A higher density of piscivorous individuals could lead to and contribute to strong year‐class strength, as this intra‐annual cohort can better cope with winter starvation and size‐related predation mortality. On the other hand, a lower density of piscivorous pikeperch could lead to weaker year‐class strength and ultimately prevent the potential role of pikeperch in regulating fish density in the food web and influencing ecological dynamics (Leeuwen et al. 2014).

Ontogenetic diet shifts in fish play a crucial role in shaping ecological dynamics by enabling resource partitioning and promoting the coexistence of sympatric predatory species (Huss et al. 2013; Sánchez‐Hernández 2019; Westrelin et al. 2023). As previous studies have shown, predatory fishes like YOY pikeperch, perch, and northern pike initially feed on zooplankton, resulting in direct competition with each other and numerous other planktivores. However, as they grow, their diet shifts to macroinvertebrates and fish, leading to a reduction in the overlap between each other. Furthermore, the diet and habitat shift leads to a redistribution of predation pressure across different prey groups and habitats, contributes to an efficient transfer of biomass and energy, increases the stability of the trophic web, and supports the resilience of the ecosystem (Caskenette and Mccann 2017; Sánchez‐Hernández 2019).

## 5 Conclusion

This study provides empirical evidence for the two distinct ontogenetic dietary shifts and isotopic niche partitioning between YOY pikeperch conspecifics and their crucial role in shaping population and ecological dynamics. Previous studies documented the discontinuous size distribution of YOY pikeperch in some years in temperate lakes or reservoirs and explained the possible mechanisms beyond the occurrence of discontinuous size distribution. However, our study combined the results of GCA and SIA and provided direct evidence that a broad size range of young of the year, driven by the variation in the diet sources, resulted in trophic niche partitioning between intra‐annual cohorts as a vital response to intraspecific competition for resources and facilitates coexistence and promotes individual specialization. Finally, it is important to note that there may be variations between years in diet and isotopic position among YOY intra‐annual cohorts. Also, the presence of three distinct intra‐annual cohorts is not always guaranteed. This particular year provided a unique opportunity to investigate diet and isotopic partitioning among the three intra‐annual cohorts of pikeperch.

## Author Contributions

**Million Tesfaye:** conceptualization (lead), data curation (lead), formal analysis (lead), investigation (supporting), methodology (lead), software (lead), validation (lead), visualization (lead), writing – original draft (lead), writing – review and editing (lead). **Allan T. Souza:** conceptualization (supporting), formal analysis (supporting), supervision (lead), validation (supporting), visualization (supporting), writing – review and editing (supporting). **Marek Šmejkal:** conceptualization (supporting), formal analysis (supporting), methodology (supporting), software (supporting), validation (supporting), writing – review and editing (supporting). **Tomáš Jůza:** investigation (lead), writing – review and editing (supporting). **Zuzana Sajdlová:** investigation (supporting), writing – review and editing (supporting). **Vladislav Draštík:** investigation (supporting), writing – review and editing (supporting). **Luboš Kočvara:** investigation (equal), methodology (equal), writing – review and editing (supporting). **Radka Symonová:** investigation (supporting), writing – review and editing (supporting). **Travis B. Meador:** data curation (supporting), formal analysis (lead), methodology (supporting), validation (lead), writing – review and editing (supporting). **Mojmír Vašek:** conceptualization (equal), data curation (equal), formal analysis (lead), investigation (equal), methodology (lead), software (equal), validation (lead), writing – review and editing (equal). **Jan Kubečka:** conceptualization (equal), funding acquisition (lead), investigation (lead), methodology (lead), project administration (lead), resources (lead), software (equal), supervision (lead), validation (supporting), writing – review and editing (supporting).

## Conflicts of Interest

The authors declare no conflicts of interest.

## Supporting information


**Data S1:** ece371973‐sup‐0001‐DataS1.xlsx.

## Data Availability

All the required data are uploaded as Data S1.
